# Evaluating the causal association between bronchiectasis and different types of inflammatory bowel disease: a two-sample Mendelian randomization study

**DOI:** 10.3389/fimmu.2024.1365108

**Published:** 2024-04-04

**Authors:** Qian Zeng, Da Hu, Yuan Li, Zhiwei Zhou, Jinfeng Wu, Xiaodong Li, Xiqiu Yu

**Affiliations:** ^1^ Department of General Practice, Shenzhen Luohu Hospital Group Luohu People’s Hospital, The Third Affiliated Hospital of Shenzhen University, Shenzhen, Guangdong, China; ^2^ Department of Gastroenterology, Shenzhen Luohu Hospital Group Luohu People’s Hospital, The Third Affiliated Hospital of Shenzhen University, Shenzhen, Guangdong, China

**Keywords:** inflammatory bowel disease, Crohn’s disease, ulcerative colitis, bronchiectasis, mendelian randomization, causal relationship

## Abstract

**Background and objectives:**

Previous observational studies have established a connection between bronchiectasis and inflammatory bowel disease (IBD), but none of these studies have provided a clear explanation for the underlying cause of this relationship. The present study thus implemented Mendelian randomization (MR) design to explore possible bidirectional relationships between IBD and bronchiectasis risk, with an additional focus on Crohn’s disease (CD) and ulcerative colitis (UC) as IBD subtypes.

**Materials and methods:**

A large genome-wide association study (GWAS)-derived data pool was leveraged to examine the relationships between bronchiectasis and IBD, CD, and UC. Two-sample MR analyses were performed with an inverse variance weighted (IVW) approach supplemented with the MR-Egger and weighted median methods. Sensitivity analyses were used to further assess the reliability of the main MR study findings. The possibility of reverse causation was also evaluated using a reverse MR approach.

**Results:**

The IVW MR analytical approach revealed that IBD (p = 0.074), UC (p = 0.094), and CD (p = 0.644) had no significant impact on the incidence of bronchiectasis, with the converse also being true (p = 0.471, p = 0.700, and p = 0.099, respectively).

**Conclusion:**

This MR analysis demonstrated that the higher occurrence of bronchiectasis in patients with IBD is not caused by genetic predisposition.

## Introduction

Inflammatory bowel disease (IBD) is a term that refers to a condition characterized by the autoimmune-mediated, nonspecific, chronic inflammation of the gastrointestinal tract that can be further classified into Crohn’s disease (CD) and ulcerative colitis (UC). In clinical settings, IBD patients experience an array of debilitating symptoms including nausea, diarrhea, abdominal pain, bloody stool, and anemia (1). IBD is characterized by a relapsing-remitting course that can lead to serious complications such as strictures, fistulas, infections, and cancer. It is estimated that the number of cases of IBD worldwide was about 3.32 million in 1990, while it has increased by 47.45% in 20 years to reach 4.9 million cases by 2019. As a result, the disease places an enormous burden on society, the economy, and healthcare (2). IBD can additionally result in the incidence of a variety of extra-intestinal adverse events impacting the genitourinary, hepatobiliary, skeletal muscle, ocular, cutaneous, and pulmonary systems (3). These complications affect anywhere from 21-47% of patients with IBD (4, 5).

One of the most common pulmonary consequences of IBD that has been identified is bronchiectasis, which affects UC patients more commonly than CD patients (6, 7). Such lung involvement is also more often observed in females relative to males (8). Bronchiectasis is a pathological condition characterized by the irreversible dilation of the bronchi, resulting in symptoms including chronic sputum production, coughing, and more frequent pulmonary infections or exacerbations thereof (9). The common embryonic foregut origin of the lung and gut is believed to contribute to the detrimental pulmonary outcomes observed in patients with IBD. Additionally, the lung-gut axis, which refers to the bidirectional influence between the lung and intestine through microbial composition and immune response, also plays a significant role in this phenomenon. Alterations in intestinal microorganisms may affect the development and progression of bronchial asthma and chronic obstructive pulmonary disease (COPD) via the lung-gut axis (10–12). Conversely, allergens abnormally activated in lung inflammation or immune responses can impair lung function while simultaneously triggering the production of inflammatory substances in the intestines (13). Previous research has indicated that acute lung injury disrupts the pulmonary microbiota, leading to transient bacterial translocation into the bloodstream and an acute increase in intestinal bacterial load (14).

Evidence from reviews and prospective studies suggests a potential bidirectional relationship between bronchiectasis and IBD; however, the underlying mechanisms are yet to be elucidated (15, 16). The increased risk of pulmonary involvement in IBD has been primarily linked to factors such as IBD-related surgery (17), opportunistic infections, immunosuppressive therapies, and drug toxicity, or it may be directly associated with the inflammation inherent to IBD itself (18). Given that these reports on IBD and bronchiectasis are observational, this hampers efforts to simultaneously consider all interventional strategies (immunosuppressive agents, rectal resection surgery), socioeconomic factors (family support, education, stress, etc.), behavioural factors (exercise, diet, sleep, etc.), and environmental factors (air pollution, climate). Exploring the potential causal relationship between IBD and bronchiectasis is further complicated by the possibility of confounding interactions between these variables. In the realm of genetic inheritance, the research conducted by Papanikolaou et al. has indicated that various extra-intestinal manifestations of IBD show genetic predispositions: specific haplotypes, including HLA-A2 and HLA-DR1 in CD and HLA-B27, HLA-B58, and HLA-B8/D3 in UC, have been found to have strong associations with joint, skin, and ocular diseases (4). However, there has yet to be a clear demonstration of a specific genetic predisposition for respiratory involvement. It is therefore plausible to hypothesize that the genetic susceptibility for bronchiectasis may be linked to an increased risk of IBD. Early identification of the relationship between IBD and airway involvement is crucial for specialists in pulmonology, internal medicine, and gastroenterology, as these conditions typically respond positively to inhaled steroid therapy (19, 20). Consequently, elucidating the directional and causal relationship between IBD and bronchiectasis represents a pressing need in the medical community.

Therefore, this article utilizes the Mendelian Randomization (MR) approach, leveraging publicly available data from large-scale Genome-Wide Association Studies (GWAS) that include lineage-specific genetic variations as instrumental variables (IVs). This approach is employed to investigate the causal relationships between exposure and outcome phenotypes. By employing random allocation to assign participants to different treatment and control groups across two independent cohorts, this study aims to mitigate confounding factors and reverse causality effects. **Table 1** offers detailed information on the case and control cohorts. For the first time, this provides a reliable genetic-level elucidation that there is no potential bidirectional causal relationship between IBD and bronchiectasis. This insight holds significant implications for optimizing the clinical management of subclinical bronchiectasis in patients with IBD, thereby reducing the risk of incorrect and ineffective treatments and preventing irreversible changes in the airways.

**Table 1:** Data sources.

Disease	Data source	GWAS ID	Cases/Controls
IBD	https://gwas.mrcieu.ac.uk/datasets/ieu-a-31/	ieu-a-31	12,882/21,770
CD	https://gwas.mrcieu.ac.uk/datasets/ukb-a-552/	ukb-a-552	732/336,467
UC	https://gwas.mrcieu.ac.uk/datasets/ebi-a-GCST90038684/	ebi-a-GCST90038684	2,515/482,083
Bronchiectasis	https://gwas.mrcieu.ac.uk/datasets/finn-b-J10_BRONCHIECTASIS/	finn-b-J10_BRONCHIECTASIS	1,107/186,723

GWAS, Genome-Wide Association Studies; IBD, Inflammatory bowel disease; UC, Ulcerative Colitis; CD, Crohn’s Disease.

## Materials and methods

### Study design

In this study, we utilized a two-sample MR framework based on summary-level data, deriving estimates of the effects of genetic variations on exposure and outcomes from two separate datasets. The study is divided into two main parts: (i) exploring bronchiectasis as the exposure and IBD, including CD and UC, as the outcomes; and (ii) examining IBD as the exposure and bronchiectasis as the outcome. Our analyses followed three critical steps to ensure methodological rigor: 1). Compliance with three foundational assumptions of the MR approach to minimize bias and reinforce the robustness of our findings. Initially, we applied a significance threshold of p < 5 × 10^-8^. If exposed single nucleotide polymorphisms (SNPs) fell short of the minimum count of 10 required for MR studies (21, 22), we adopted a less stringent threshold of p < 5 × 10^-6^ (23, 24), satisfying our first hypothesis. To address the remaining hypotheses, Linkage Disequilibrium (LD) Clumping (r² < 0.001, distance = 10,000 kb) was performed on all IVs to reduce SNP correlation effects. Horizontal pleiotropy was evaluated using MR-Egger regression, the weighted median method, and leave-one-out analysis. 2) Examination of causal relationships employing five standard MR analysis techniques under varied assumptions. 3) Consideration of multiplicity and heterogeneity, coupled with performing sensitivity analyses to confirm the reliability of our results. As this research was conducted using publicly available data and aggregated statistical analyses, no specific ethical approval or informed consent was necessary. The R code used for our analyses is provided in **Supplementary Figures 1**-**6**.

### Data sources and instruments

#### Bronchiectasis

Summary statistics related to bronchiectasis were obtained from a prior GWAS of individuals of European ancestry (data link: https://gwas.mrcieu.ac.uk/datasets/finn-b-J10_BRONCHIECTASIS/), enabling the identification of bronchiectasis-related SNPs using p < 5 × 10^-8^ as a selection threshold. As only a single bronchiectasis-associated SNP exhibited a p-value below this threshold, the cut-off was extended to p < 5×10^-6^, excluding any SNPs in linkage disequilibrium (within 5000 kb or *r^2^* > 0.01). Based on this approach, 17 independent bronchiectasis-related SNPs were identified, all of which were used as IVs for UC, while 14 and 16 were used as IVs when analyzing IBD and CD, respectively. The diagnosis of bronchiectasis is based on the ICD-10 (International Classification of Diseases) criteria.

#### IBD data

The summary statistics for IBD were sourced from a GWAS meta-analysis involving 12,716,084 SNPs conducted by the International Inflammatory Bowel Disease Genetics Consortium (IIBDGC), which comprised 12,882 cases and 21,770 controls (data link: https://gwas.mrcieu.ac.uk/datasets/ieu-a-31/). The genetic association data available for this study consisted of analyses of 484,598 patients with UC (N=2515 cases, 482,083 controls) and 337,199 patients with CD (N=732 cases, 336,467 controls), respectively covering 9,587,836 and 10,894,596 SNPs in UC and CD. The patients were of European descent, and their diagnoses had been made using accepted standards for radiological, endoscopic, and histological assessment. Since the whole basis of these studies was drawn from publically accessible data and pooled statistical analyses, the current study did not require any particular ethical review or informed permission.

Individual and cumulative F-statistics for each SNP were computed as follows to provide light on the possible influence of weak instrument bias on estimated effect sizes for the relevant causal relationship: F = R2 × (N - 2)/(1 - R2), where R2 corresponds to the variance in the exposure explained by each IV. Those IVs that exhibited an F-statistic < 10 were regarded as weak instruments such that they were not included when performing MR analyses (25).

### Statistical analysis

R (v 4.3.1) was used to conduct MR analyses with the MR-PRESSO (version 1.0) and Two Sample MR packages (version 0.5.6). The inverse variance weighted (IVW), weighted median, weighted mode, and MR-Egger regression procedures were among the supplementary methods used to estimate the causal relationships between exposures and outcomes. Of these, the IVW method was the main analytical technique used to look at connections between CD, UC, or IBD and bronchiectasis. For assessing result heterogeneity, leave-one-out SNP analysis and Conchrane’s Q test were used. Sensitivity analyses were also employed to assess the consistency and robustness of these results with the weighted median, MR-PRESSO, and MR-Egger regression approaches. Using a significance threshold of p < 0.05, the intercept term obtained from the MR-Egger regression was employed to evaluate the possibility of directional pleiotropy. Potential horizontal pleiotropy was identified by evaluating the asymmetry of the funnel plot. The MR-PRESSO test was utilized to assess the presence of pleiotropy bias and adjust for its influence by eliminating potential outliers. A reverse MR analysis was then performed to test for any possible reverse causality between IBD (UC, CD) and bronchiectasis. The “leave-one-out” analysis was conducted by sequentially eliminating one SNP at a time to mitigate bias caused by the horizontal pleiotropy of individual SNPs.

## Results

### The causal impact of bronchiectasis on IBD, CD, and UC

**Figure 1:**
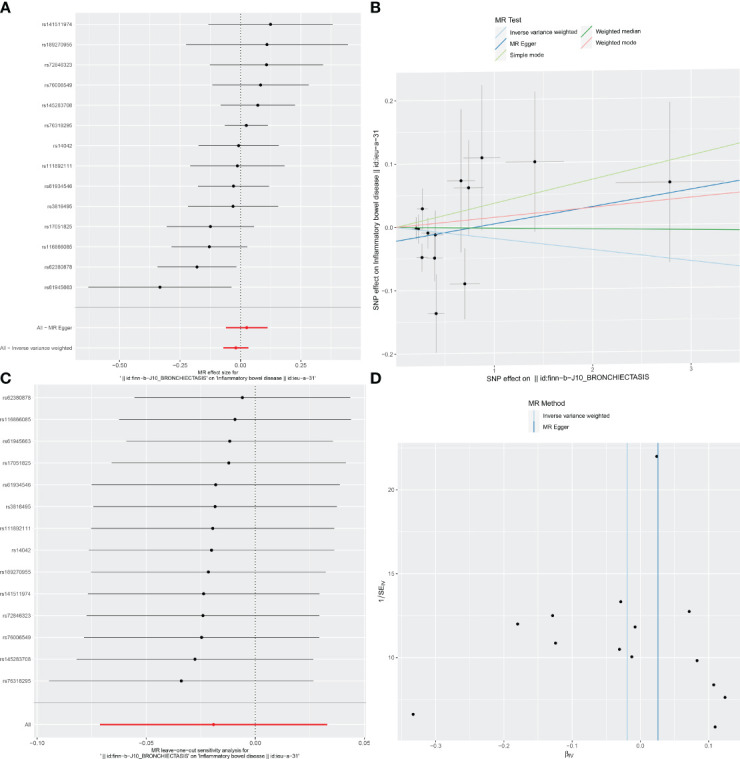
Visualizations of the causal impact of bronchiectasis on IBD risk. **(A)** Forest plots; **(B)** Scatter plots; **(C)** Leave-one-out analyses; **(D)** Funnel plots. IBD, Inflammatory bowel disease.

**Figure 2:**
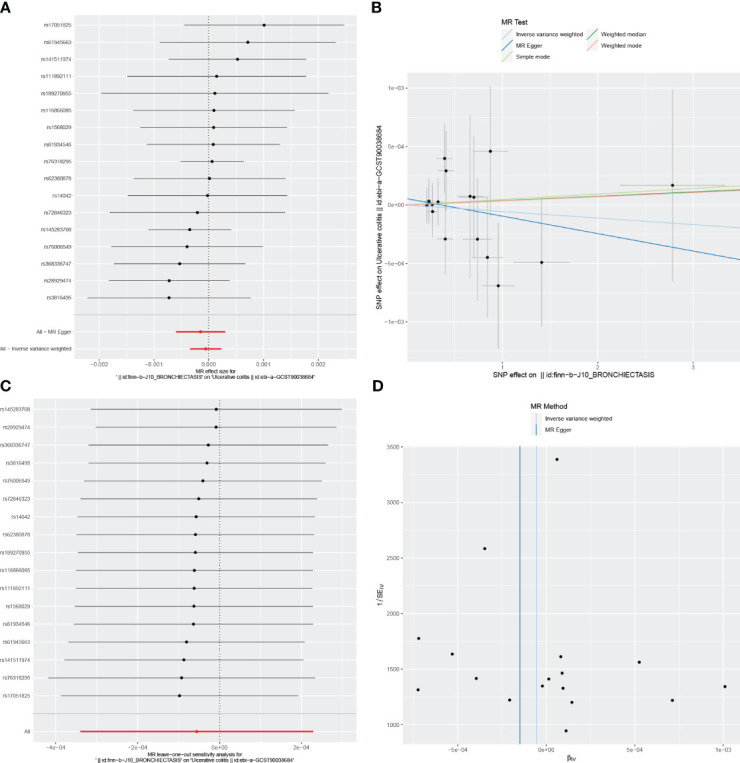
Visualizations of the causal impact of bronchiectasis on UC risk. **(A)** Forest plots; **(B)** Scatter plots; **(C)** Leave-one-out analyses; **(D)** Funnel plots. UC, Ulcerative Colitis.

**Figure 3:**
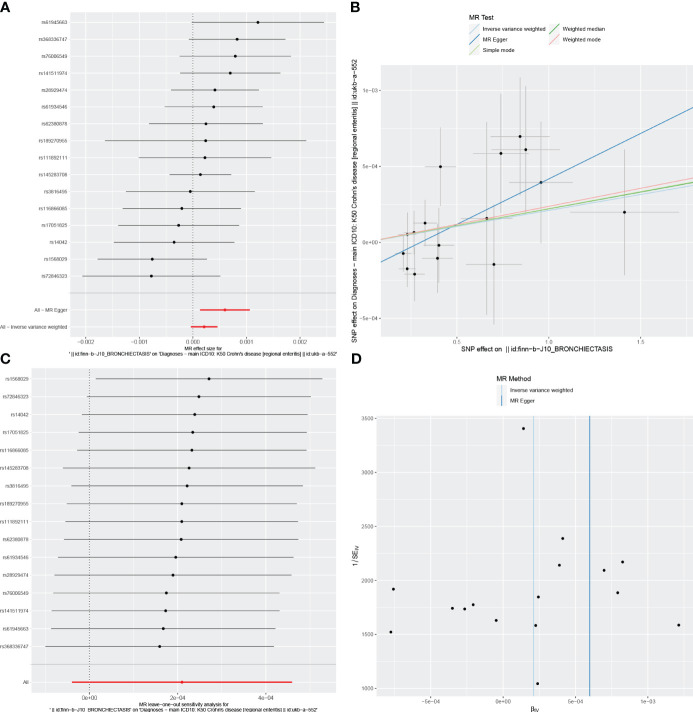
Visualizations of the causal impact of bronchiectasis on CD risk. **(A)** Forest plots; **(B)** Scatter plots; **(C)** Leave-one-out analyses; **(D)** Funnel plots. CD, Crohn’s Disease.

For these analyses, fewer than 10 SNPs for bronchiectasis as exposures were identified at a significance threshold of p < 5 × 10^-8^. When using a broader threshold of p < 5 × 10^-6^, we identified 14, 17, and 16 independent SNPs related to bronchiectasis from GWAS. These SNPs were selected as IVs for analyzing the risk of IBD, UC and CD, respectively (**Supplementary Table 1**-**3**). All of these IVs exhibited F-statistic values greater than 20. MR analysis results performed using different methods are summarized in **Table 2**. In summary, these studies failed to uncover any evidence of a causal connection between the genetically predicted risk of bronchiectasis and IBD. IVW results indicated that there was no significant correlation between an increase in bronchiectasis risk and the odds of developing IBD (IBD: OR = 0.981, 95% CI: 0.931-1.033, *p* = 0.471; UC: or =0.999, 95% CI 0.999- 1.002, *p* = 0.7003; CD: or =1.000, 95% CI: 0.9996-1.0005, *p* = 0.099), with similar results stemming from the weighted median, weighted mode, and MR-Egger analyses. In summary, these studies failed to uncover any evidence of a causal connection between the genetically predicted risk of bronchiectasis and IBD. **Figures 1**, **2**, **3A** and **1**-**3B** visualize the MR results through forest plots and scatter plots, respectively. Leave-one-out analyses indicated that the causal estimates pertaining to the association between bronchiectasis and IBD subtypes were not driven by any individual SNP (**Figures 1**-**3C**), while MR-Egger intercept test results failed to yield any evidence of directional horizontal pleiotropy (**Figures 1**-**3D**).

**Table 2:** Two-sample MR estimates for the effect of Bronchiectasis on IBD and Subtype (UC and CD).

Exposure	Outcome	Method	OR	95%CI	*P*-value	Heterogeneity	Pleiotropy
Bronchiectasis	IBD	MR Egger	1.026	0.941-1.118	0.5688	0.2229	0.2299
		Weighted median	0.997	0.934-1.065	0.9356		
		IVW	0.981	0.931-1.033	0.4713	0.1819	
		Simple mode	1.037	0.934-1.148	0.4957		
		Weighted mode	1.014	0.940-1.095	0.7223		
Bronchiectasis	UC	MR Egger	0.999	0.9994-1.0003	0.524	0.943	0.605
		Weighted median	1	0.9996-1.0004	0.8555		
		IVW	0.999	0.9997-1.0002	0.7003	0.956	
		Simple mode	1	0.9994-1.0007	0.885		
		Weighted mode	1	0.9995-1.0006	0.8931		
Bronchiectasis	CD	MR Egger	1	1.0001-1.0011	0.024	0.662	0.0731
		Weighted median	1	0.9998-1.0006	0.23		
		IVW	1	0.99996-1.0005	0.0994	0.448	
		Simple mode	1	0.99958-1.00086	0.519		
		Weighted mode	1	0.9997-1.0008	0.342		

OR, odds ratio; CI, confidence interval; Heterogeneity, p value for Cochran’s Q test; Pleiotropy, p value for MR-Egger intercept test; IBD, Inflammatory bowel disease; UC, Ulcerative Colitis; CD, Crohn’s Disease; IVW, Inverse Variance Weighted.

### Reverse MR analyses

**Figure 4:**
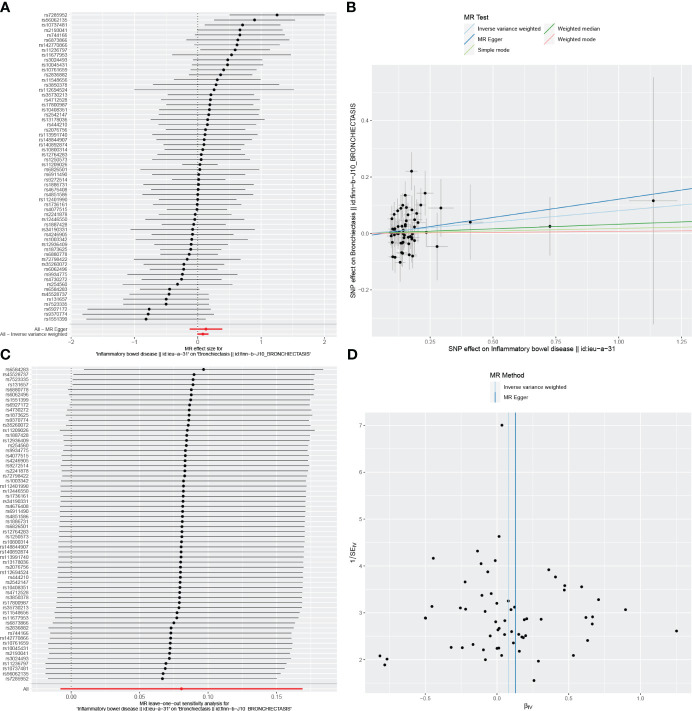
Visualizations of the causal impact of IBD on Bronchiectasis risk. **(A)** Forest plots; **(B)** Scatter plots; **(C)** Leave-one-out analyses; **(D)** Funnel plots. IBD, Inflammatory bowel disease.

**Figure 5:**
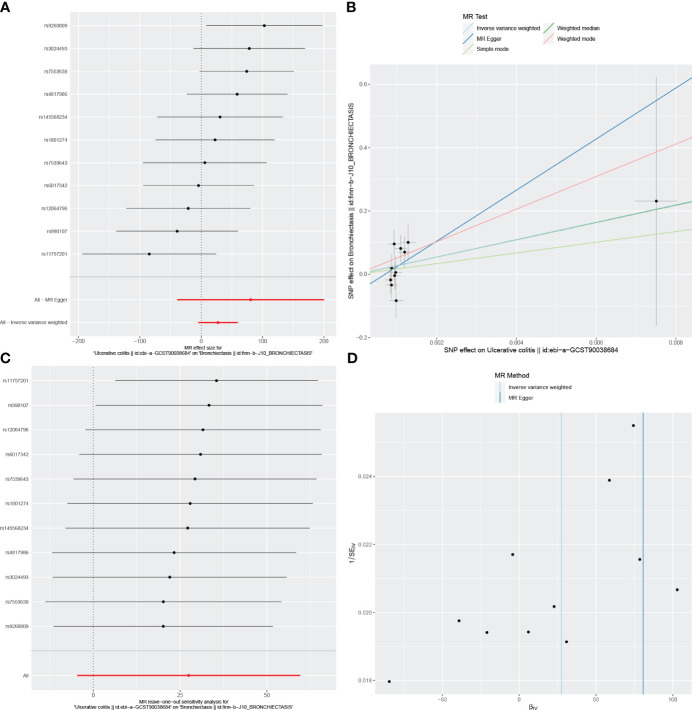
Visualizations of the causal impact of UC on Bronchiectasis risk. **(A)** Forest plots; **(B)** Scatter plots; **(C)** Leave-one-out analyses; **(D)** Funnel plots. UC, Ulcerative Colitis.

**Figure 6:**
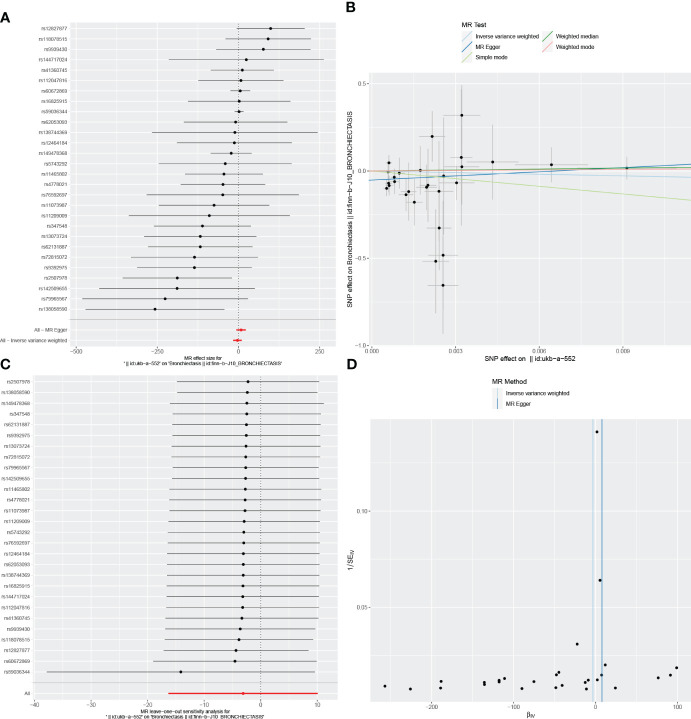
Visualizations of the causal impact of CD on Bronchiectasis risk. **(A)** Forest plots; **(B)** Scatter plots; **(C)** Leave-one-out analyses; **(D)** Funnel plots. CD, Crohn’s Disease.

Reverse MR analyses were also used to test the possible effects of IBD, UC, or CD on the incidence of bronchiectasis. Using the IVW method, no evidence of a reverse causal relationship among these conditions was detected (**Table 3**). Results for other sensitivity methods are presented in **Figures 4**–**6**.

**Table 3:** Two-sample MR estimates for the effect of IBD and Subtype (UC and CD) on Bronchiectasis.

Exposure	Outcome	method	OR	95%CI	*P*-value	Heterogeneity	Pleiotropy
IBD	Bronchiectasis	MR Egger	1.1383	0.879-1.472	0.3279	0.139	0.693
		Weighted median	1.033	0.910-1.173	0.6133		
		IVW	1.084	0.992-1.184	0.074	0.155	
		Simple mode	1.018	0.795-1.303	0.888		
		Weighted mode	1.007	0.834-1.217	0.94		
UC	Bronchiectasis	MR Egger	1.08E+35	1.122E-17~1.036E+87	0.219	0.22	0.389
		Weighted median	7.11E+11	6.583E-06~7.678E+28	0.173		
		IVW	8.54E+11	9.603E-03~7.593E+25	0.0936		
		Simple mode	2.16E+07	1.907E-24 ~2.438E+38	0.653	0.226	
		Weighted mode	2.22E+22	2.295E-07~ 2.148E+51	0.162		
CD	Bronchiectasis	MR Egger	2.75E+03	1.062E-03~ 7.132E+09	0.303	0.344	0.181
		Weighted median	6.38E+00	8.585E-06~4.737E+06	0.788		
		IVW	4.45E-02	8.136E-08~2.429E+04	0.644	0.131	
		Simple mode	4.46E-07	1.484E-29~1.340E+16	0.57		
		Weighted mode	2.81E+00	1.077E-05~7.321E+05	0.876		

OR, odds ratio; CI, confidence interval; Heterogeneity, p value for Cochran’s Q test; Pleiotropy, p value for MR-Egger intercept test; IBD, Inflammatory bowel disease; UC, Ulcerative Colitis; CD, Crohn’s Disease; IVW, Inverse Variance Weighted.

In the reverse MR analysis, we identified 62 and 11 SNPs (thresholds used were p < 5 × 10^-8^), and 27 SNPs (thresholds used were p < 5 × 10^-6^) for testing the possible effects of IBD, UC, or CD on the incidence of bronchiectasis, respectively (**Supplementary Table 4**-**6**). The F-statistics for all SNPs were greater than 10, suggesting no evidence of weak instrumental variables. In the primary IVW MR analysis, we found no association between IBD, UC, CD and bronchiectasis (IBD: IVW: OR = 1.084, 95% *CI* = 0.992-1.184, *p*-value = 0.074, UC: IVW: OR = 8.54E+11, 95% *CI* = 9.603E-03-7.593E+25, *p*-value = 0.094; CD: IVW: OR = 4.45E-02, 95% *CI* = 8.136E-08~2.429E+04, *p*-value = 0.644), nor did the other methods (including MR-Egger regression and weighted median) (all *p* > 0.05) (**Table 3**). Forest plots were generated to display the effects of individual SNPs and the aggregated effect of all SNPs (**Figures 4**-**6A**). Scatter plots compared the estimates from different MR methods, demonstrating general consistency across methodologies (**Figure 4**-**6B**). Sensitivity analyses, which involved excluding individual SNPs one at a time, showed that no single SNP significantly altered the overall results (**Figures 4C**, **5C**, **6C**). Additionally, funnel plots indicated visual symmetry, suggesting the absence of directional horizontal pleiotropy (**Figures 4D**, **5D**, **6D**).

## Discussion

This study is the first to investigate the cause-and-effect relationship between genetic vulnerability to IBD and the risk of bronchiectasis. The researchers used a variety of complementary methodologies known as MR to examine this correlation. The bidirectional Mendelian randomization analyses finally did not find any indication of a causal relationship between genetically predicted IBD and bronchiectasis in individuals of European descent. Similarly, in investigating the correlation between respiratory diseases and IBD, Freuer and colleagues identified no significant association between the genetically predicted prevalence of adult asthma and IBD using MR. This observation aligns with the findings from our own MR analysis (26).

In observational studies, bronchiectasis is generally regarded as the most common form of pulmonary complication among IBD patients (7, 16, 27, 28). The first report of bronchopulmonary involvement was published by Kraft et al. in 1976, when they identified 6 IBD patients exhibiting large volumes of bronchial secretions of unknown origin, with or without bronchiectasis (29). Desai et al. conducted a prospective analysis of pulmonary function tests (PFTs) and high-resolution computed tomography (HRCT) scans of the chest in patients with IBD. They discovered a higher prevalence of bronchiectasis in IBD patients compared to controls, although the majority of these patients exhibited no clinical symptoms (15). Jiang et al. explored the genetic correlation between three pulmonary function traits and four gastrointestinal diseases. While epidemiological studies suggested a protective effect of pulmonary function against gastrointestinal diseases, no significant causal relationship between pulmonary function and gastrointestinal conditions was established through bidirectional MR analysis (30). Some evidence suggests that IBD-related bronchiectasis can respond well to the administration of inhaled corticosteroids (31, 32). The pathogenic basis for pulmonary involvement in IBD patients may be attributable to the shared embryonic origin and immunological similarity between the bronchial and intestinal mucosa (33). However, it remains poorly understood as to whether bronchiectasis typically manifests prior to or following the diagnosis of IBD, and some guidelines do not consider IBD as a relevant background or causal factor associated with pulmonary involvement (34). We believe that the discrepancies between the estimates from observational reports and our findings may stem from the inherent differences between the two analytical approaches. Observational studies are inevitably susceptible to clinical confounding factors, which can affect both exposure and outcomes. Although these studies may indicate a strong association between the two, they cannot establish a direct causal relationship. Due to these factors, the causal relationship between IBD and bronchiectasis remains to be clarified, and the genetic basis for susceptibility related to respiratory involvement has not yet been determined. MR can mitigate the impact of these confounding factors by utilizing genetic instrumental variables. Especially for investigating bidirectional causal relationships, MR remains a reliable method for testing hypothesized effects.

The present MR analysis results do not support any causal relationship between IBD and bronchiectasis in either direction from a genetic perspective, suggesting that yet-to-be-determined confounding factors likely influence this relationship. Notably, bronchiectasis incidence has been reported in patients with both CD and UC following colon resection surgery (17), and colectomy may induce or worsen these symptoms (29). Smoking has been identified as a protective factor for UC that is significantly negatively correlated with a history of prior colon resection surgery and more severe colon inflammation (34). Due to the prevalence of systemic health concerns in people with IBD, observational studies are unable to investigate causal or reverse links between IBD and bronchiectasis. IBD is a systemic condition that commonly shows extraintestinal manifestations affecting various systems in the body. These include the skin (pyoderma gangrenosum, erythema nodosum), hepatobiliary system (primary sclerosing cholangitis, hepatitis, pancreatitis, portal vein thrombosis), musculoskeletal system (arthritis), genitourinary system (renal calculi, obstructive nephropathy), and ocular system (uveitis, conjunctivitis, scleritis). These disorders might increase the production of inflammatory mediators, especially when combined with opportunistic infections. This can potentially influence the connection between IBD and bronchiectasis (18, 27, 35). Our study establishes that there is no genetic causality between IBD and bronchiectasis, leading us to posit that the connection between gastrointestinal disorders and lung function is more correlative than causative. Consequently, attention should shift towards exploring other potential shared mechanisms or risk factors. This includes considering the impact of chronic inflammation or immune system abnormalities in the comprehensive treatment and management of patients.

In clinical practice, physicians may not necessarily need to be overly concerned about one disease being a direct result of the other when evaluating treatment strategies and preventive measures for patients with either of these conditions. Nevertheless, it is essential to recognize that lung function serves as a crucial indicator of pulmonary health. Prioritizing lung function assessment can facilitate the early detection of changes indicative of lung pathology and aid in the formulation of effective interventions. Furthermore, the findings from our study underscore the significance of accounting for individual differences when devising treatment plans. Given the lack of a direct link between the two diseases, physicians should perhaps focus more on a patient’s personal medical history and genetic predisposition rather than formulating a treatment strategy based on a presumed association between these diseases.

There are some limitations to these analyses. Firstly, the study population was exclusively composed of individuals of European ancestry. Given that the distribution of specific genetic markers or variants may vary among different ethnicities, this could influence disease susceptibility and outcomes. Therefore, future research should encompass a more diverse range of populations to ensure the widespread applicability and validation of our findings. Moreover, as detailed outpatient information was unavailable for study subjects, subgroup analyses could not be performed to gain more granular insight into these potential causal relationships. Although we implemented multiple measures to avoid potential confounders and related horizontal pleiotropy, we cannot completely rule out the impact of horizontal pleiotropy, as the biological functions of many genetic variations remain unclear. Lastly, MR studies are situated between interventional and observational studies in the evidence period such that they fail to provide evidence as strong as that derived from randomized clinical trials (RCTs) or systematic reviews thereof (36).

In summary, the present results did not reveal any evidence of a causal association between genetically predicted bronchiectasis and IBD. Given the importance of identifying the factors underlying airway involvement in IBD patients for internists, pulmonologists, and gastroenterologists, additional large-scale GWAS meta-analyses and studies utilizing alternative genetic tools will be vital to validate and build upon the present results. To further dissect the complex relationship between bronchiectasis and IBD, additional research based on diverse population backgrounds and experimental studies is necessary. Utilizing independent experimental data for functional characterization, and validation in separate cohorts, are essential steps to enhance the clinical significance of our research findings. This approach will pave the way for personalized medicine and improve management strategies for patients with these complex conditions.

## Data availability statement

The original contributions presented in the study are included in the article/**Supplementary Materials**, further inquiries can be directed to the corresponding author/s.

## Ethics statement

Ethical approval was not required for the studies involving humans because the studies were conducted in accordance with the local legislation and institutional requirements. The human samples used in this study were acquired from https://gwas.mrcieu.ac.uk/datasets/. Written informed consent to participate in this study was not required from the participants or the participants’ legal guardians/next of kin in accordance with the national legislation and the institutional requirements.

## Author contributions

QZ: Writing – review & editing, Writing – original draft. DH: Writing – review & editing. YL: Writing – review & editing, Data curation. ZZ: Writing – original draft. JW: Writing – review & editing. XL: Writing – review & editing. XY: Writing – review & editing.
