# Supplementary figures and images for: Evaluating the causal association between bronchiectasis and different types of inflammatory bowel disease: a two-sample Mendelian randomization study
Source: Front Immunol. 2024 Apr 4;15:1365108. doi: 10.3389/fimmu.2024.1365108 (PMC11024297; doi:10.3389/fimmu.2024.1365108)

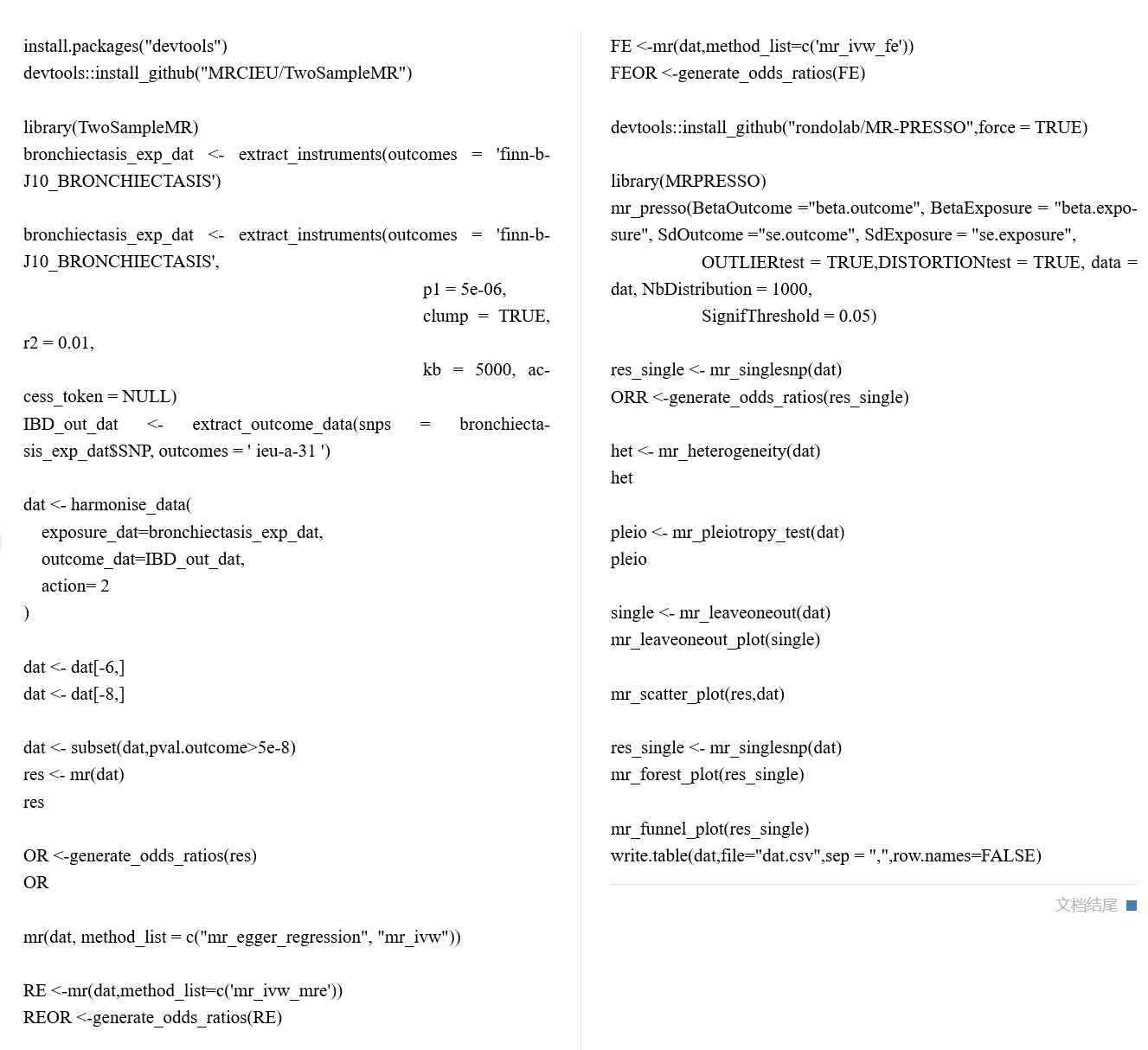

Supplement: Supplementary file 1 [file Image_1.jpeg]

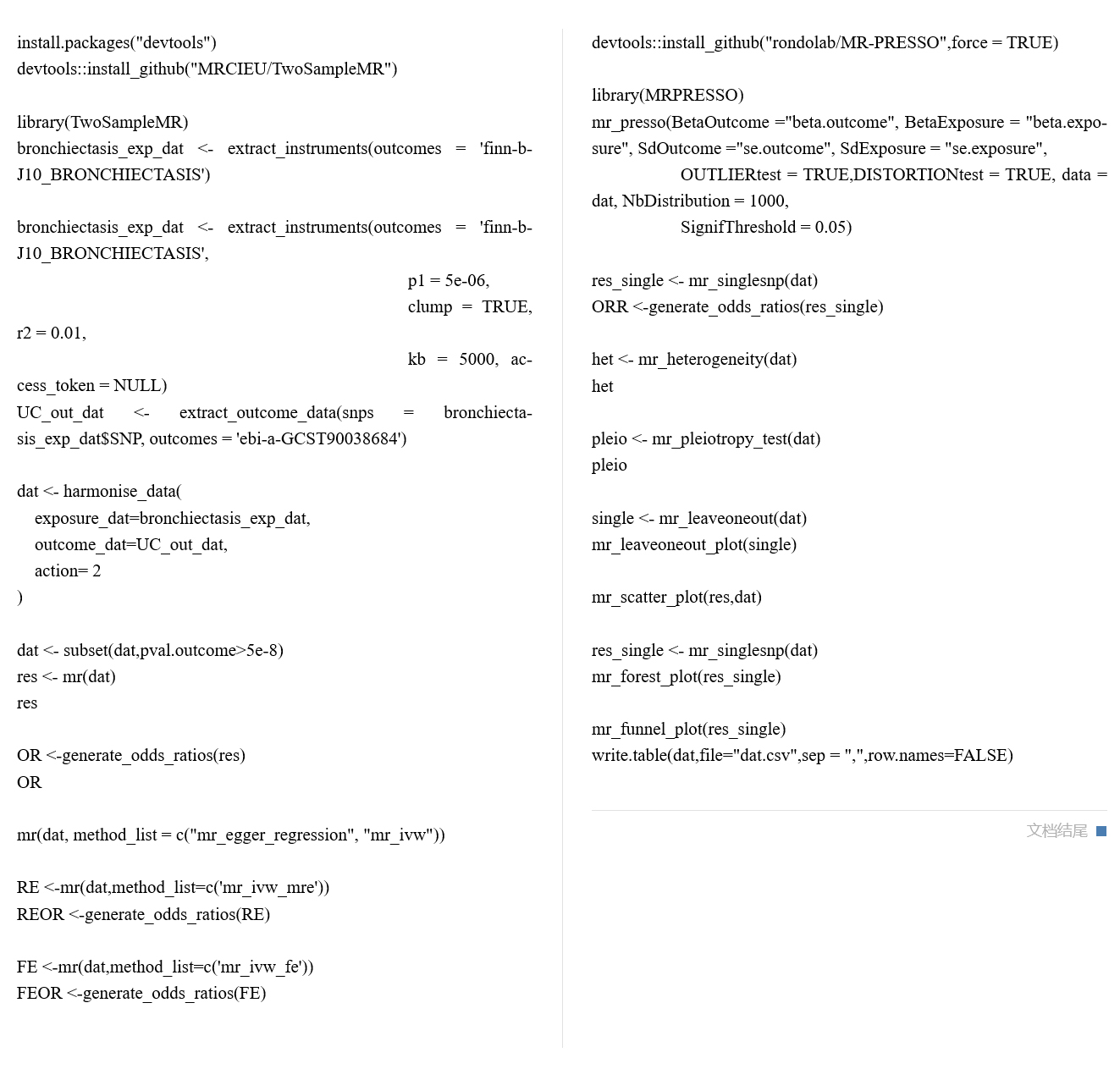

Supplement: Supplementary file 2 [file Image_2.jpeg]

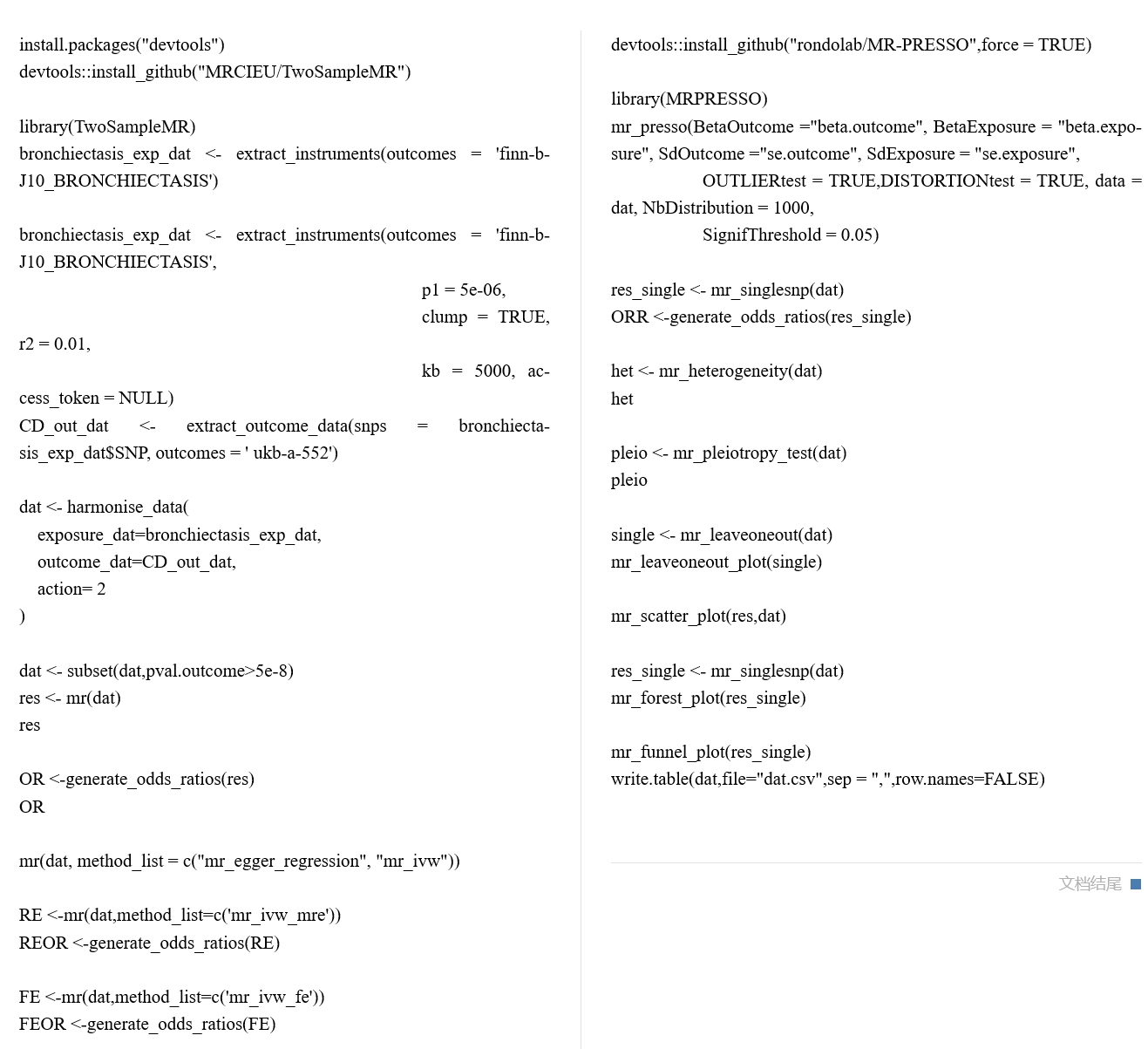

Supplement: Supplementary file 3 [file Image_3.jpeg]

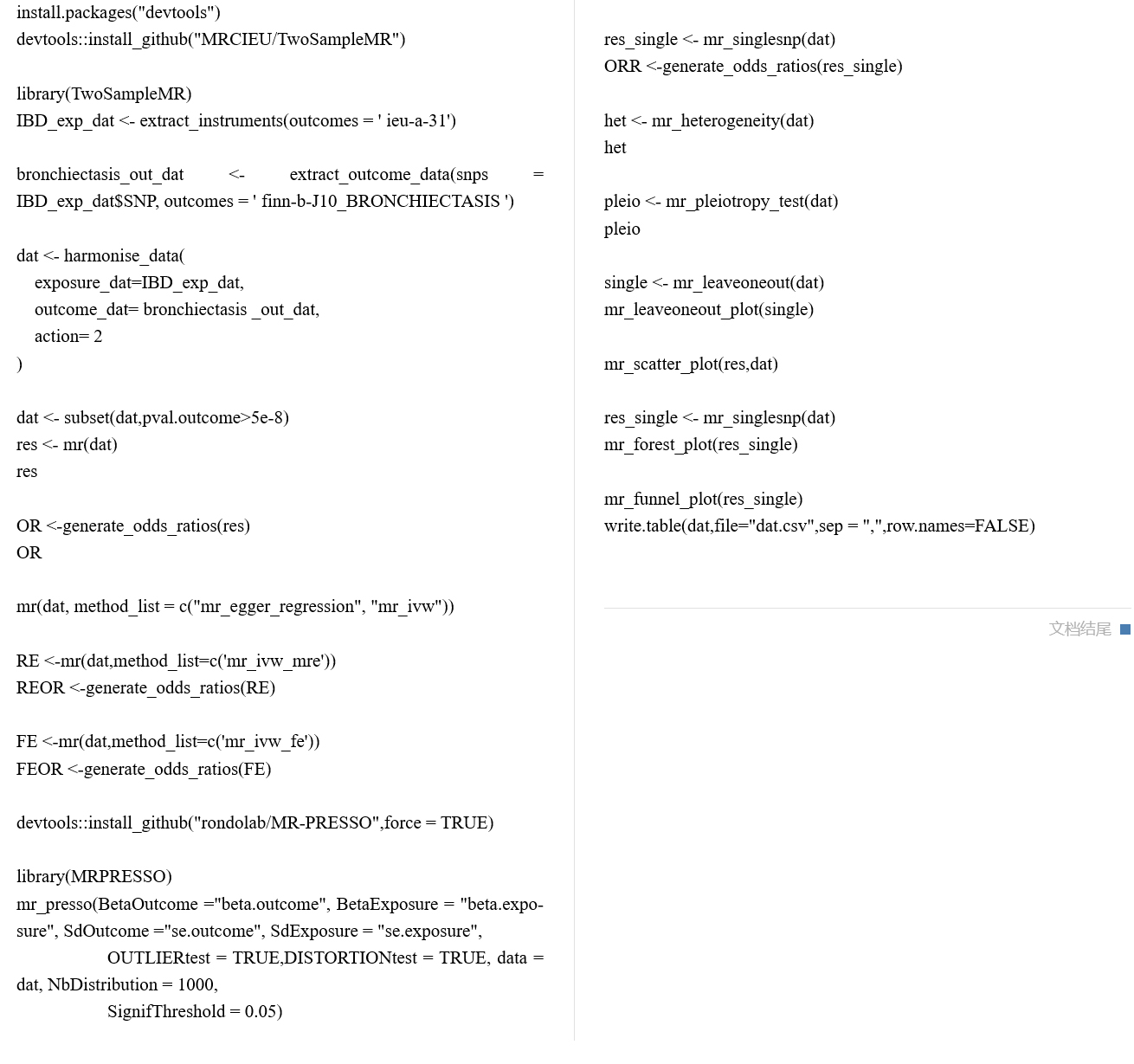

Supplement: Supplementary file 4 [file Image_4.jpeg]

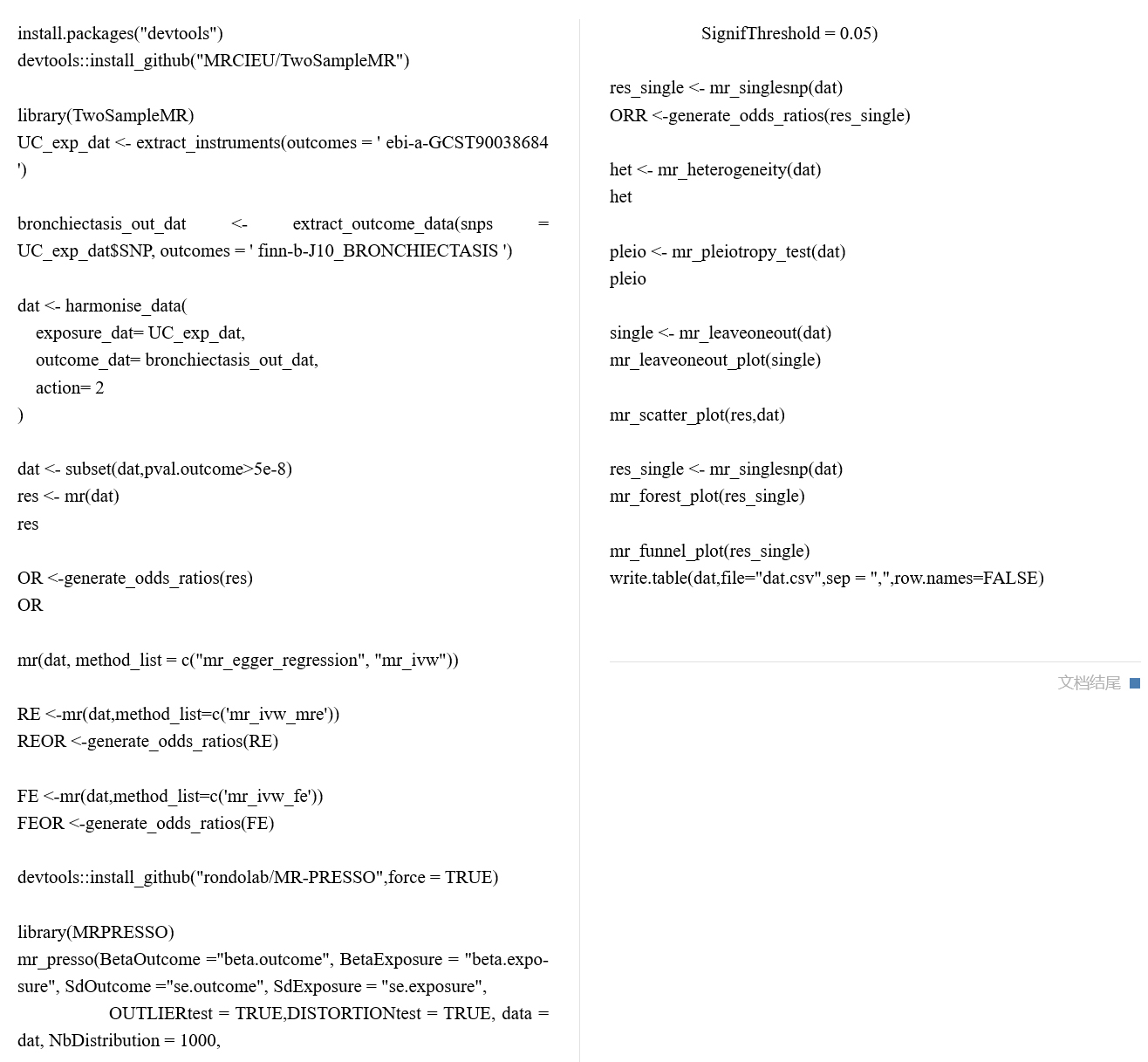

Supplement: Supplementary file 5 [file Image_5.jpeg]

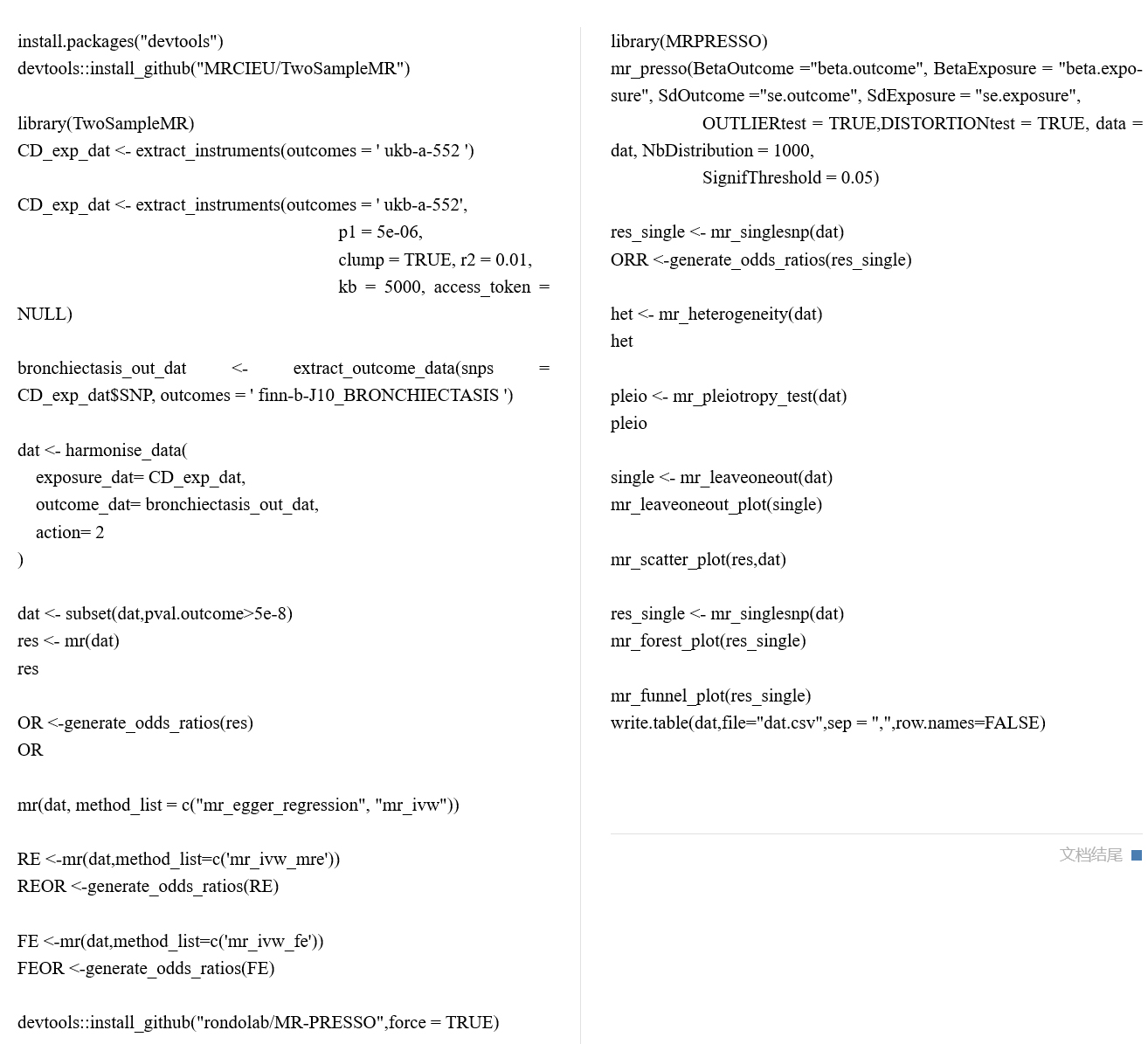

Supplement: Supplementary file 6 [file Image_6.jpeg]
